# Determinants of dropout in a community-based mental health crisis centre

**DOI:** 10.1186/s12888-016-0819-4

**Published:** 2016-04-19

**Authors:** Alexandre Henzen, Clotilde Moeglin, Panteleimon Giannakopoulos, Othman Sentissi

**Affiliations:** Mental Health and Psychiatry Department, University Hospitals of Geneva, CAPPI Jonction: 35, rue des Bains, 1205 Geneva, Switzerland; Psychiatric Department, University Hospitals of Geneva, Chemin du Petit-Bel-Air 2, CH- 1225 Chêne-Bourg, Switzerland; Catchment Area and Mental Health Units, Mental Health and Psychiatric Department, University Hospitals of Geneva, CAPPI Jonction: 35, rue des Bains, 1205 Geneva, Switzerland

**Keywords:** Dropout, Mentally ill patient, Attachment, Crisis centre

## Abstract

**Background:**

Dropping out during the course of medical follow up is defined as an early therapy withdrawal without the agreement of the therapist. In a psychiatric crisis unit in Geneva, we empirically observed that almost 50 % of the patients were not showing up to their first appointments, which were scheduled for 3 to 7 days post discharge.

**Methods:**

The aim of this naturalistic descriptive cohort study is to identify the demographic, patient and care-related predictive factors of dropout in a community-based psychiatric crisis centre. We included 245 consecutive outpatients followed-up for 4 to 6 weeks of intensive outpatient psychiatric treatment. Logistic regression models were built to examine the association between dropout and demographic, care and patient-related variables.

**Results:**

Among the 245 outpatients, dropout occurred in 37.5 % of cases, and it most frequently occurred (81.8 %) in the first 2 days of follow-up. Among care-related variables, referral by hospital units or private psychiatrists led to significantly lower levels of dropout compared to patients referred by the psychiatric emergency unit (respectively: OR = .32; *p* = .04; 95 % CI [.10, .93]; OR = .36; *p* = .04; 95 % CI [.13, .96]; OR = .22; *p* = .002; 95 % CI [.08, .58]). Among patient-related variables, younger age increased the risk of dropout (OR = .96; 95 %; *p* = .002; 95 % CI [.94, .99]). Anxiety and personality but not mood disorders were also related to higher rates of dropout (respectively: OR = 2.40; *p* = .02; 95 % CI [1.14, 4.99]; and OR = 1.98; *p* = .02; 95 % CI [1.09, 3.59]). Unipolar depression (72.2 %; OR = 1.47; p = .48; 95 % CI [.34, 1.21]) was the most frequent primary diagnosis in this sample.

**Conclusions:**

This study makes clear the need for increased efforts to improve care adherence in young patients with anxious or personality disorders seen in emergency rooms because they are prone to early discontinuation of treatments. Future studies in this field are warranted to gain a better understanding into the complex reasons that surround discontinuation of care in outpatient settings.

## Background

The rate of dropping out of treatment in psychiatric outpatients is highly variable and depends on the definition and time of drop out as well as the characteristics of the population sample and study design [1, 2]. Estimated prevalence varies from 15 to 60 % [3–6] and is even higher among young people [4, 7–9], particularly in the age group of 15–24 years [4]. Kolb et al. [10] defined dropout as missing two consecutive sessions; Hatchett et al. [11] defined it as not attending the last scheduled session; Longo et al. [12] defined it as not returning after a preliminary interview; and Pekarik [13] defined it as cessation of therapy without the agreement of the therapist, regardless of the number of sessions. Dropout is more frequent for initial appointments [14]. In 2008, Barett and coll. [15] reported that among 100 patients in a care centre, only half of them would return after a first assessment, no more than one-third would return after the first therapy session, only 20 of them would come to the third meeting, and under 17 would complete more than 10 sessions. In a cohort of 349 patients from a community mental health centre, Salta and Buick [16] reported that the dropout rate decreased when the patients continued attending after the third meeting.

The relationship between mental illness and dropout is still a matter of debate. Personality disorders are one of the main predictive factors of dropout [17]. In an early study, Aapro et al. [18] showed that patients with addiction, antisocial or impulsive personality traits tended to discontinue therapy early. According to Ogrodniczuk and Piper [19], patients with borderline personality disorder are more likely to discontinue early in analytic therapy. Schizophrenia could also increase the risk of drop out [20]. De Panfilis et al. [21] showed that a history of suicide attempts predicted early discontinuation of therapy, whereas the presence of an eating disorder and avoidant personality features were negatively associated with early dropout.

Demographic determinants of dropout are still unclear. Pierzbicki and Pekarik [22] reported no association between demographic factors and dropout. Other studies found an association between dropping out of treatment and time from first contact [17], low income and the lack of insurance coverage [4], living alone, and low socioeconomic status [20]. A prospective study of 365 patients observed that patients who miss psychiatric follow-up are more severely ill and poorly socially functioning than those who attend their appointments [23].

To address the demographic, patient-related and care related determinants of dropout, we performed a naturalistic study in a psychiatric crisis unit in Geneva, a second-line multidisciplinary care centre for patients with acute psychiatric disorders requiring intensive treatment. Among the care-related predictors, delay between referral and first consultation (less or more than 24 h) and referral type were considered to explore whether treatment timeliness and the referring provider impact on dropout occurrence in this community-based setting [24–29].

## Method

The study was conducted in the crisis unit of one of the four public psychiatry centres of the University Hospitals of Geneva. These psychiatric and psychotherapeutic ambulatory centres cover a catchment area of 120,000 inhabitants and provide both conventional day care models and crisis intervention. The Central Ethics Committee of the University Hospitals of Geneva approved the project. According to the rules of the local Ethics Committee, the use of anonymized data for quality control in clinical divisions is authorized without individual written consent. The present study did not involve any additional investigation or invasive procedures as a part of the general authorization for retrospective studies in the division of general psychiatry. All experiments were in compliance with the Helsinki declaration.

The study included 245 consecutive outpatients followed-up over 4 to 6 weeks of intensive psychiatric treatment. To be close to a real life situation, we considered all psychiatric diagnoses. Only patients with major physical problems (including cardiovascular disease, cancer or central nervous system pathologies), sensory deficits, or follow-up patients from the community mental health consultation were excluded from the study. The clinical diagnoses were made by two independent board certified psychiatrists according to the ICD-10 criteria.

### Measures

The dropout criteria included either initial non-attendance for the first scheduled appointment (initial dropout) and care discontinuation after the first or the second consultation (secondary dropout). If the follow up reached 7 days, early discontinuation was not considered as dropout. The intensity of monitoring thereafter averaged 3 times per week. Clinical diagnosis was established following the ICD-10 criteria by two independent and fully trained psychiatrists. Doubtful cases (cases in which there were disagreement between the two clinicians) were excluded from the present study. In addition to clinical diagnosis, the following demographic variables were explored as predictors of dropout in regression models: age, gender, treating psychiatrist (presence/absence), Swiss origin, Swiss status, marital status (married), education (master’s degree, apprenticeship, or compulsory education), and current activity (employee, unemployed, or pensioner). Among care-related predictors, delay between referral and first consultation (less or more than 24 h) and referral type (emergency, crisis hospital unit, hospital, general physician, psychiatrist, specialized program, addictive program, or themselves) were taken into account. Among patient-related variables, the presence or absence of past psychiatric history, current psychiatric diagnosis, number of psychiatric hospitalizations, substance abuse comorbidities according to the ICD-10 criteria, psychotropic treatment (antidepressants, benzodiazepines, neuroleptics) and suicide thoughts (presence or absence at inclusion) were considered.

### Statistical analyses

Comparisons of demographic, care-related and patient-related variables between the care group and dropout group were conducted using the Chi Square test for categorical variables and analysis of variance (ANOVA) for continuous variables.

The Kolmogorov-Smirnov test was used to ensure the normality of the distribution. We built univariate logistic regression models to check the association between each demographic, patient and care-related predictor (independent variable) with dropout (yes/no) as the dependent variable. Multivariate regression models were built using only the independent variables that significantly predicted dropout in univariate models. In addition, a one-way analysis of variance was used to assess the differences in dropout frequencies between 5 age groups (less than 30 years, from 30 to 40, from 40 to 50, from 50 to 60, and more than 60 years). Statistical analyses were performed using SPSS for PC (version 19 Chicago, Illinois, USA) software. The level of significance was *p* < 0.05, and the results are reported as averages.

## Results

### Demographic variables

The mean age of the population studied was 41 years old (18–67, 39 % men). Among these patients, 67 % lived alone, 38 % had no or basic education and 63 % were unemployed or received invalidity pensions. Fifty per cent of the patients were followed-up by a private psychiatrist or psychologist (Table 1). Fifty-five per cent of the patients were referred by units of the psychiatric department (of which 22.5 % were from the emergency room and 9.4 % were from the hospital crisis unit), 20.1 % came by themselves, 16.4 % came from a private psychiatrist and 8.2 % were referred by general physicians. Furthermore, 77.6 % of patients had a history of psychiatric disorders, and 34.7 % had been hospitalized at least once (Table 2).

**Table 1:** Demographic characteristics of patients and univariate logistic regression model for predictors of dropout concerning 245 patients

	Total n (%)	*b* (SD)	Wald	Df	*P* valeur	Odds ratio	95 % CI
Age (mean ± sd)	41.14 (11.79)	−.04 (.01)	10.30	1	.002	.96	.94 to .99
Gender							
Male	95 (38.8)	−.35 (.28)	1.60	1	.21	.71	.41 to 1.22
Female	150 (61.2)					1	
Treating psychiatrist	121 (49.4)	−.31 (.27)	1.37	1	.24	.73	.43 to 1.24
Swiss origin	102 (41.6)	−.46 (.27)	2.83	1	.09	.63	.37 to 1.08
Swiss status	144 (58.8)	−.51 (.27)	3.57	1	.06	.60	.35 to 1.02
Marital status (married)	80 (32.7)	−.41 (.29)	2.00	1	.16	.67	.37 to 1.18
Education			3.32	2	.19		
Master’s degree	47 (19.2)	−.70 (.39)	3.25	1	.07	.50	.23 to 1.07
Apprenticeship	106 (43.3)	−.28 (.29)	.92	1	.34	.76	.42 to 1.34
Compulsory education	92 (37.6)					1	
Current activity			.71	2	.70		
Employee	91 (37.1)	.01 (.40)	>.001	1	.99	1.01	.46 to 2.2
Unemployed	114 (46.5)	.23 (.38)	.36	1	.55	1.26	.59 to 2.66
Pensionner	40 (16.3)					1

**Table 2:** Clinical characteristics of patients and univariate logistic regression model for predictors of dropout concerning 245 patients

	Total n (%)	*b* (SD)	Wald	Df	*P* valeur	Odds ratio	95 % CI
Care-related variables							
Referral type			15.44	7	.03		
Emergency	55 (22.5)					1	
Crisis hospital unit	23 (9.4)	−1.15 (.55)	4.44	1	.04	.32	.10 to .93
Hospital	28 (11.5)	−1.03 (.50)	4.24	1	.04	.36	.13 to .96
General physician	20 (8.2)	−.73 (.54)	1.81	1	.18	.48	.16 to 1.40
Psychiatrist	40 (16.4)	−1.50 (.48)	9.76	1	.002	.22	.08 to .58
Specialized program	14 (5.7)	−1.03 (.65)	2.49	1	.12	.36	.10 to 1.29
Addictive program	15 (6.1)	.02 (.58)	.002	1	.97	1.03	.32 to 3.22
Themselves	49 (20.1)	−.31 (.39)	.63	1	.43	.73	.33 to 1.59
Change of referents	45 (18.4)	−.59 (.43)	1.87	1	.17	.56	.24 to 1.28
Delay < 24 h	119 (48.6)	.02 (.27)	.01	1	.93	1.02	.60 to 1.72
Patient-related variables							
Psychiatric history							
With Psy Atcd	190 (77.6)	−.13 (.31)	.18	1	.67	.88	.47 to 1.62
Consultation	14 (5.7)	−.43 (.61)	.50	1	.48	.65	.19 to 2.14
Hospitalization	85 (34.7)	.08 (.28)	.09	1	.76	1.09	.63 to 1.87
BTC therapy	81 (33.1)	−.11 (.28)	.16	1	.69	.89	.51 to 1.56
Psychiatrist	120 (49.0)	−.21 (.27)	.65	1	.42	.81	.48 to 1.36
Specialized program	16 (6.5)	−.002 (.53)	>.001	1	.997	.998	.35 to 2.85
Addiction unit	14 (5.7)	−.43 (.61)	.50	1	.48	.65	.19 to 2.14
Diagnostics							
Disorders due to psychoactive susbtance use	74 (30.2)	.02 (.30)	.004	1	.95	1.02	.58 to 1.79
Schizophrenia, schizotypal personality and delusional disorders	22 (9.0)	−.28 (.48)	.34	1	.56	.76	.29 to 1.94
Mood disorders	195 (79.6)	−.44 (.32)	1.90	1	.17	.64	.34 to 1.21
Unipolar	177 (72.2)	.39 (.55)	.50	1	.48	1.47	.50 to 4.32
Anxiety, dissociative, stress-related, somatoform and other nonpsychotic mental disorders	34 (13.9)	.87 (.37)	5.44	1	.02	2.40	1.14 to 4.99
Disorders of adult personality and behavior	60 (24.5)	.69 (.30)	5.16	1	.02	1.98	1.09 to 3.59
Dependence syndrome					.15		
Yes	65 (26.5)	.49 (.29)	2.78	1	.10	1.63	.91 to 2.91
No	180 (73.5)					1	
Past suicidal thgt (yes)	104 (42.4)	.21 (.27)	.62	1	.43	1.23	.73 to 2.08
History of suicidal attempt (yes)	65 (26.5)	−.41 (.31)	1.72	1	.19	.67	.36 to 1.22
Psychotropic treatments (yes)	173 (70.6)	−.49 (.29)	2.96	1	.09	.61	.34 to 1.08
Antidepressant	117 (47.8)	−.63 (.27)	5.51	1	.02	.53	.31 to .91
Benzodiazepines	106 (43.3)	−.64 (.27)	5.44	1	.02	.53	.30 to .91
Antipsychotics	71 (29.0)	−.51 (.30)	2.79	1	.09	.60	.33 to 1.10

Legend: *Psy Atcd* Psychiatric antecedent; *Delay < 24 h* delay before starting the care in the centre inferior of 24 h, *Past suicidal thgt* Past suicidal thoughts, *BTC* Brief therapy centres

### Clinical characteristics

Unipolar depression (72.2 %) was the most frequent primary diagnosis in this sample, followed by substance- induced disorders (30.2 %) and personality disorder (24.5 %). Only 9 % of the population had a diagnosis of schizophrenia, schizotypal personality disorder, delusional disorder and other non-mood psychotic disorders (F20-29). Psychotropic medication was prescribed in 70.6 % of the patients (mostly antidepressant treatment, in 47.80 % of cases). Among the referral characteristics of the cohort, half of the population was evaluated less than 24h after referral (Table 2).

### Dropout

**Fig. 1:**
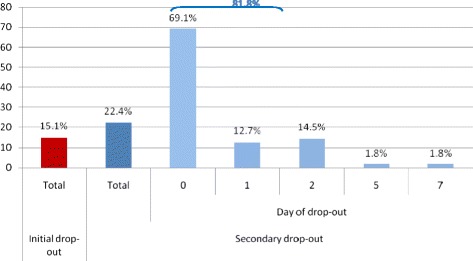
Characteristics of patients (*N* = 245) concerning initial and secondary dropout, and the day the dropout occurred. Legend: Initial dropout: initial non-attendance for the first scheduled appointment. Secondary dropout: care discontinuation after the first or the second consultation

Dropout occurred in 37.5 % of cases and most frequently (81.8 %) within the first 2 days of follow-up (secondary dropout, see Fig. 1). There were no statistically significant differences in the main demographic, care-related and patient-related variables between initial and secondary dropout groups.

### Univariate regression analysis

Among care-related variables, referrals from the hospital crisis unit, the psychiatric hospital, or by private psychiatrists were associated with lower rates of dropout than patients referred by the psychiatric emergency services (22.5 %) (respectively: OR = .32; *p* = .04; 95 % CI [.10, .93]; OR = .36; *p* = .04; 95 % CI [.13, .96]; OR = .22; *p* = .002; 95 % CI [.08, .58]). Patients who started their care in the centre within 24 h did not have a significantly greater amount of dropouts than patients seen later on (Tables 1 and 2).

Among patient-related variables, younger age raised the risk of dropout (OR = .96; 95 %; *p* = .002; 95 % CI [.94, .99]). >One-way analysis showed that the significant contrasts concern the “less of 30 years” group compared with “50 to 60” and “more than 60 years” groups (respectively: *t* (96.75) = 2.38, *p* = .020; *t* (27.97) = 2.41, *p* = .023). Other contrasts were not non-significant. Moreover, both anxiety and personality disorders were related to increased rates of dropout (respectively: OR = 2.40; *p* = .02; 95 % CI [1.14, 4.99]; and OR = 1.98; *p* = .02; 95 % CI [1.09, 3.59]), but mood disorders had no impact on dropout rates (see Table 2). The dropout rate was not significantly influenced by the patient’s psychiatric history, past suicide thoughts, past history of suicide attempts, or substance abuse-related disorders. Interestingly, patients who were taking an antidepressant or benzodiazepine were less likely to drop out than patients who were not using these treatments (OR = .53; *p* = .02; 95 % CI [.30, .91] for both treatments). There was no effect of gender, education, or other demographic variables on the risk of dropout.

### Multivariate regression analysis

For the multiple regression analysis, significant variables (*p* < .05) in the univariate logistic regression analysis were taken into account**.** Only referral by private psychiatrists was associated with lower rates of dropout, compared to patients sent by psychiatric emergencies (OR = .26; *p* = .005; 95 % CI [.09, .67]).

## Discussion

Our data show that dropout in an outpatient crisis centre may be determined by a combination of demographic, clinical and care-related parameters. In particular, our results reveal that being younger, referral from psychiatric emergencies and presence of anxiety or personality disorders predict a more frequent rate of dropout occurring both before the first consultation and in the two first days of the follow-up.

Overall, there was a 37.5 % rate of dropout in the present sample, a percentage in line with those reported previously in community-based outpatient settings [3–6, 30]. Unlike in previous reports, most demographic variables in our study were not related to dropout. Bueno Heredia et al. [20] reported that living alone, being divorced, unmarried or widowed, and a low socioeconomic status were predictive of dropout. A low level of education was also a predictive factor of dropout in two meta-analyses [22, 23]. In our study, education level was not associated with increased dropout risk, possibly because of the relative rarity of patients with a high school education. Interestingly, previous psychiatric history did not decrease this risk, suggesting that being aware of one’s vulnerability does not necessarily lead to better adherence to treatment. This is in contrast with past findings, in which previous treatment has been associated with a lower dropout rate [31, 32].

Younger age was the only significant predictive factor of dropout we found, in line with previous observations in this field [4, 7–9, 30]. Adolescence and young adulthood is a period of identity crisis associated with a risk of loss of control and conflict, especially for a psychotherapist, who can be seen as a parental figure.

As is often the case, 81.8 % of the dropout in our study occurred in the first 2 days of medical follow-up [14–16]. It is likely that once the medical follow up is organized, dropout is rare, possibly due to an increase of the therapeutic alliance: patients who did not feel they established a positive relationship with the therapist are unlikely to come back and they may end therapy prematurely [33].

Importantly, the diagnoses of anxiety disorder and personality disorder were significant predictors of dropouts. It is possible that this is due to the difficulty of anxious patients to overcome their fears or for the emotionally labile patients to maintain stable interpersonal bonds [18, 19, 21]. In 2012, Martino et al. showed that both borderline personality disorders and subjective experience (motivation, treatment expectation, therapeutic relation perception and barriers to access) predict premature termination of treatment. Moreover, patients with borderline personality disorder who experienced a less satisfactory therapeutic relationship and reported many external problems were more likely to drop out of the programme [17]. In contrast to the common thought, substance abuse and psychosis were not associated with increased dropout rate in this study. Despite the poor compliance often reported in psychotic patients [20], Rossi et al. stressed that in a community-based psychiatric service targeted to patients with severe mental illness, schizophrenia was a “perfect” predictor for not dropping out [34]. It is possible that the multidisciplinary approach of our crisis centre, which includes both nurse and social worker interventions, may prevent a substantial number of dropouts in the case of patients with addiction and psychotic disorders. Reneses et al. [3] reported that the influence of a particular practitioner as well as the centre’s care-setting may be important factors in discontinuation. Antidepressant and anxiolytic treatments could be protective factors in preventing drop out of patients suffering from anxiety disorders.

The patients who were referred to the crisis programme by the emergency units are prone to significantly more dropout than patients sent by psychiatrist crisis hospital unit and hospital units, private settings or patients who came on their own. Furthermore, the multivariate logistic regression indicates that referral from private psychiatrists led to significantly lower levels of dropout compared to patients referred by the psychiatric emergency unit. Taken together these data suggest that a special attention should be given to young patients with anxiety and personality disorders who are first assessed in emergency settings since they are more exposed to dropout.

Strengths of the present study are the inclusion of all psychiatric morbidities as well as careful documentation of demographic parameters and care-related variables. Among its limitations are the lack of clinical data for initial non attendance patients and their social functioning, absence of structured interviews to sustain the clinical diagnosis made by two independent psychiatrists and inclusion of cases in a crisis management setting that are not representative of the whole spectrum of patients followed-up in community-based facilities.

## Conclusion

This study shows the need for increased efforts to improve care adherence in young patients with anxious or personality disorders observed in emergency rooms because they are prone to early discontinuation of treatments. Future studies in this field addressing these limitations are warranted to gain a better understanding into the complex reasons that surround discontinuation of care in outpatient settings.

### Open access

This article is distributed under the terms of the Creative Commons Attribution 4.0 International License (http://creativecommons.org/licenses/by/4.0/), which permits unrestricted use, distribution, and reproduction in any medium, provided you give appropriate credit to the original author(s) and the source, provide a link to the Creative Commons license, and indicate if changes were made. The Creative Commons Public Domain Dedication waiver (http://creativecommons.org/publicdomain/zero/1.0/) applies to the data made available in this article, unless otherwise stated.

### Ethics and consent to participate statement

The Central Ethics Committee of the University Hospitals of Geneva approved the project. According to the rules of the local Ethics Committee, the use of anonymized data for quality control in clinical divisions is authorized without individual written consent. The present study did not involve any additional investigation or invasive procedures as a part of the general authorization for retrospective studies in the division of general psychiatry. All experiments were in compliance with the Helsinki declaration.

### Consent for publication

Not applicable.

### Availability of data and materials statement

All data supporting our findings will be shared on request.

## References

[1] Sentissi O, Bartolomei J, Moeglin C, Baeriswyl-Cottin R, Rey-Bellet P (2014). The Geneva model of crisis intervention. A retrospective study. J Psychol Psychother.

[2] Huisman S, Maes S, De Gucht V, Chatrou M, Haak H (2010). Low goal ownership predicts dropout from a weight intervention study in overweight patients with type 2 diabetes. Int J Behav Med.

[3] Reneses B, Munoz E, Lopez-Ibor JJ (2009). Factors predicting dropout in community mental health centres. World Psychiatry.

[4] Edlung MJ, Wang PS, Berglund P, Katz SJ, Lin E, Kessler RC (2002). Dropping out of mental health treatment: patterns and predictors among epidemiological survey respondents in the United States and Ontario. Am J Psychiatry.

[5] Centorrino F, Hernan MA, Drago-Ferrante G, Rendall M, Apicella A, Länger G, Baldessarini RJ (2001). Factors associated with noncompliance with psychiatric outpatient visits. Psychiatr Serv.

[6] Pang AH, Lum FC, Ungvari GS, Wong CK, Leung YS (1996). A prospective outcome study of pagtients missing regular psychiatric outpatient appointments. Soc Psychiatry Psychiatr Epidemiol.

[7] Pelkonen M, Marttunen M, Laippala P, Lönnqvist J (2000). Factors associated with early dropout from adolescent psychiatric outpatient treatment. J Am Acad Child Adolesc Pschiatry.

[8] Armbruster P, Fallon T (1994). Clinical, sociodemographic and systems risk factors for attrition in a childrens’s mental health clinic. Am J Orthopsychiatry.

[9] Trautman PD, Stewart N, Morishima A (1993). Are adolescent suicide attempters noncompliant with outpatient care?. J Am Acad Child Adolesc Psychiatry.

[10] Kolb DL, Beutler LE, Davis CS, Crago M, Shanfield SB (1985). Patient and therapy process variables relating to dropout and change in psychotherapy. Psychothe Theor Res Pract Train.

[11] Hatchett GT, Han K, Cooker PG (2002). Predicting premature termination from counseling using the Butcher Treatment Planning Inventory. Assessment.

[12] Longo DA, Lent RW, Brown SK (1992). Social cogntive variables in the prediction of client motivation and attrition. J Couns Psychol.

[13] Pekarik G (1992). Relationship of clients’ reasons for dropping out of treatment to outcome and satisfaction. J Clin Psychol.

[14] Carpenter PJ, Morrow GR, Del Gaudio AC (1981). Who keeps the first oupatient appointment?. Am J Pychiatry.

[15] Barrett M, Chua W, Crits-Christoph P, Gibbons M, Thompson D (2008). Early withdrawal from mental health treatment: Implications for psychotherapy practice. Psychothe Theor Res Pract Train.

[16] Salta L, Buick WP (1989). Impact of organizational change on the intake, referral and treatment of outpatients at a community mental health center. J Ment Health Adm.

[17] Martino F, Menchetti M, Pozzi E, Berardi D (2012). Predictors of dropout among personality disorders in a specialist outpatients psychosocial treatment: A preliminary study. Psychiatry Clin Neurosci.

[18] Aapro N, Dazord A, Gerin P, De Coulon N, Scariati G (1994). Psychothérapie dans un centre universitaire de formation: etude des facteurs de changement. Psychothérapies.

[19] Ogrodniczuk JS, Piper WE (1999). Use of transference interpretations in dynamically oriented individual psychotherapy for patients with personality disorders. J Personal Disord.

[20] Bueno Heredia A, Cordoba Dona JA, Escolar Pujolar A, Carmona Calvo J, Rodriguez Gomez C (2001). Refusal of treatment. Actas Esp Psiquiatr.

[21] De Panfilis C, Marchesi C, Cabrino C, Monici A, Politi V, Rossi M, Maggini C (2012). Patient factors predicting early dropout from psychiatric outpatient care for borderline personality disorder. Psychiatry Res.

[22] Wierzbicki M, Pekarik G (1993). A meta-analysis of psychotherapy dropout. Prof Psychol Res Pract.

[23] Killaspy H, Banerjee S, King M, Lloyd M (2000). Prospective controlled study of psychiatric out-patient non-attendance. Br J Psychaitry.

[24] Freud S (1933). Nouvelles conférences sur la psychanalyse. AE.

[25] Freud S (2005). Le début de traitement. 1913. « Sur l’engagement du traitement », Œuvres complètes, 12.

[26] Etchegoyen H (2005). Fondements de la technique psychanalytique.

[27] Gaston L (1990). The concept of the alliance and its role in psychotherapy: theoretical and empirical considerations. Psychotherapy.

[28] Luborsky L, Cleghorn J (1976). Helping alliances in psychotherapy: the groundwork for a study of their relationship to its outcome. Successful Psychotherapy.

[29] Horvath AO, Luborsky L (1993). The role of the therapeutic alliance in psychotherapy. J Consult Clin Psychol.

[30] Swift JK, Greenberg RP (2012). Premature discontinuation in adult psychotherapy: a meta-analysis. J Consult Clin Psychol.

[31] Berghofer G, Schmidl F, Rudas S (2002). Predictors of treatment discontinuity in outpatient mental health care. Soc Psychiatry Psychiatr Epidemiol.

[32] Morlino M, Martucci G, Musella V, Bolzan M, de Girolamo G (1995). Patients dropping out of treatment in Italy. Acta Psychiatr Scand.

[33] Horvath AO, Greenberg LS (1989). Development and validation of the working alliance inventory. J Couns Psychol.

[34] Rossi A, Amaddeo F, Bisoffi G, Ruggeri M, Thornicroft G, Tansella M (2002). Dropping out of care: inappropriate terminations of contact with community-based psychiatric services. Br J Psychiatry.

